# Latent profiles of perceived social support among adolescents and their relationship with depressive symptoms

**DOI:** 10.3389/fpsyg.2025.1647562

**Published:** 2026-01-12

**Authors:** Tanming Liu, Zeng Zhou, Shiyuan Zhang, Fulan Zhang, Chuqi Yan

**Affiliations:** 1 Department of Physical Education, Central South University, Changsha, China; 2 School of Sport Science, Jishou University, Jishou, China; 3 Melbourne Graduate School of Education, University of Melbourne, Melbourne, VIC, Australia

**Keywords:** adolescents, depressive symptoms, latent profile analysis, mental health, perceived social support

## Abstract

**Objective:**

The mental health of rural adolescents is a critical public health issue, with perceived social support acting as a key buffer against depression. However, the specific patterns of this support and their differential effects remain underexplored. This study aimed to identify latent profiles of perceived social support among adolescents using latent profile analysis, examine the distribution characteristics of depressive symptoms across different profiles, and explore demographic factors influencing these profiles, providing empirical evidence for mental health interventions targeting adolescents.

**Methods:**

A stratified random cluster sampling method was used to survey 1,017 rural adolescents aged 12–18 years in the Wuling Mountain area. The Center for Epidemiologic Studies Depression Scale assessed depressive symptoms, and the Multidimensional Perceived Social Support Scale measured perceived social support. Latent Profile Analysis was conducted using Mplus 8.3 to identify latent profiles of perceived social support. Chi-square tests analyzed demographic differences across profiles, and multinomial logistic regression examined the association between perceived social support profiles and depressive symptoms.

**Results:**

Four latent profiles of perceived social support were identified: High perceived social support (43.7%), High Family Support (16.2%), Moderate perceived social support (30.4%), and Low perceived social support (9.7%). Gender and school stage significantly influenced profile distribution, with females more likely to belong to the High perceived social support (Odds Ratio = 21.76) and High Family Support (OR = 9.81) groups, and high school students more likely to fall into the Low perceived social support group. Compared with the Low perceived social support group, the Moderate group was more likely to exhibit no depression (OR = 5.81) or subthreshold depression (OR = 2.65). Both the High Family Support (OR = 8.44) and High perceived social support (OR = 4.86) groups showed significant protective effects against depression.

**Conclusion:**

Perceived social support among adolescents is heterogeneous, with family support playing a particularly strong protective role against depressive symptoms. Social support interventions should especially target male and high school student populations, offering differentiated strategies based on adolescents’ profile characteristics. Strengthening family support systems is critical for improving the mental health of rural adolescents.

## 1 Introduction

Depressive symptoms are among the most common emotional disorders in adolescents and are recognized as a major public mental health concern worldwide (McGrath et al., 2023). Based on the dimensional model of depressive symptoms, they can be classified into three stages: no depression, subthreshold depression, and clinical depression (Costello et al., 2003). Clinical depression is typically defined as a mood disorder characterized by a significant and persistent low mood resulting from various causes (Ayuso-Mateos et al., 2010). Subthreshold depression refers to a psychological subclinical state in which individuals exhibit certain depressive symptoms but do not meet the diagnostic criteria for clinical depression. It represents a transitional condition between mental health and clinical depression (Zhang et al., 2022).

Studies have shown that depressive symptoms of varying severity can exert a lasting impact on adolescents’ academic performance and social relationships. Moreover, as depressive symptoms worsen, individuals may become more prone to self-injury, suicidal behavior, or other socially harmful behaviors such as aggression or deliberate injury (Kroenke, 2017; Tuithof et al., 2018). Adolescence is a critical period for personality development and is also marked by frequent psychological conflicts. Without timely and effective intervention, these unresolved issues may lead to the onset of mental health problems or even psychiatric disorders. These outcomes not only disrupt academic and daily functioning but may also compromise physical and psychological well-being and cause serious consequences for families (Ayuso-Mateos et al., 2010; Zhao et al., 2008; Zhu et al., 2023).

Identifying protective factors for adolescent depressive symptoms is essential for early detection and prevention of mental health issues. Perceived social support—an individual’s subjective evaluation and emotional experience of the emotional or instrumental support they receive—reflects how much they feel respected, cared for, and understood within their social relationships (Hou and Chen, 2016; Norris and Kaniasty, 1996). The protective role of social support against depressive symptoms is primarily explained by two classic theoretical models: the stress-buffering hypothesis and the main effect model (Cohen and Wills, 1985). The stress-buffering hypothesis posits that social support acts as a protective shield, mitigating the negative psychological impacts of stressful life events. In the context of adolescence, stressors such as academic pressure, peer conflicts, or family discord can trigger or exacerbate depressive symptoms. Perceived social support can buffer these effects by providing emotional comfort, offering tangible assistance, and enhancing an individual’s coping resources, thereby reducing the likelihood of a stressor leading to a depressive episode (Thoits, 2011). Conversely, the main effect model suggests that social support has a direct, beneficial impact on mental health, regardless of the level of stress. By fostering a sense of belonging, stability, and self-worth, a strong support network can enhance an individual’s overall emotional well-being and resilience, making them less susceptible to depression from the outset (Rueger et al., 2016a). A substantial body of empirical evidence supports these theories, consistently demonstrating that higher levels of perceived social support are associated with a lower incidence and reduced severity of depressive symptoms in adolescents (Huang et al., 2023; Lin et al., 2024; Rueger et al., 2016a; Tian et al., 2024; Zhou et al., 2020).

Beyond these foundational models, recent research illuminates the complex interplay between social support and a wide array of psychosocial factors influencing overall well-being. Social support has been shown to be a critical mediator that can buffer the negative mental health effects of modern stressors, such as problematic social media use (Çiçek et al., 2024) and pandemic-related fears and loneliness (Koçak et al., 2025). The source of this support is paramount; positive childhood experiences and strong parental factors serve as a fundamental defense against anxiety and depression (Şanlı et al., 2024; Alshehri et al., 2020), whereas family conflict can exacerbate these risks, a relationship often mediated by the adolescent’s sense of social connectedness (Çiçek and Yıldırım, 2025). Furthermore, social support does not operate in isolation but often functions by bolstering internal resources. It can foster resilience, which in turn mediates the link between external threats like cyberbullying and psychological distress (Korkmaz et al., 2025), and also enhances psychological flexibility and self-efficacy in the face of uncertainty and future anxiety (Green et al., 2024; Öztekin et al., 2025). This protective network of support, resilience, and meaning in life collectively mitigates psychological distress and loss of motivation (Yıldırım et al., 2025; Aslan et al., 2025). Even among adults, the mental health impact of external stressors is mediated by internal cognitive styles like optimism and pessimism, which are themselves nurtured by social environments (Yıldırım and Cicek, 2022). This body of work collectively demonstrates that social support is a pivotal hub in a network of psychosocial variables that promote mental health.

### 1.1 The present study

However, most existing studies have adopted variable-centered approaches, focusing primarily on linear associations between the total score of perceived social support and depressive symptoms. This narrow focus may fail to capture the heterogeneous patterns of support adolescents perceive from different sources (e.g., family, friends, significant others) and may limit the precision and effectiveness of intervention strategies (Howard and Hoffman, 2018). To address this gap, the present study employs Latent Profile Analysis (LPA), a person-centered approach, to identify distinct subgroups of adolescents based on their patterns of perceived social support (Su et al., 2015; Wang et al., 2023; Tompson et al., 2017). By doing so, we aim to answer the following research questions:(1)What are the distinct latent profiles of perceived social support among adolescents?(2)How are adolescents with different levels of depressive symptoms (no depression, subthreshold depression, and clinical depression) distributed across these support profiles?(3) Which demographic factors predict membership in these different support profiles? Answering these questions will provide crucial empirical evidence to inform the development of more targeted and effective mental health interventions.

## 2 Materials and methods

### 2.1 Participants

**Figure 1:**
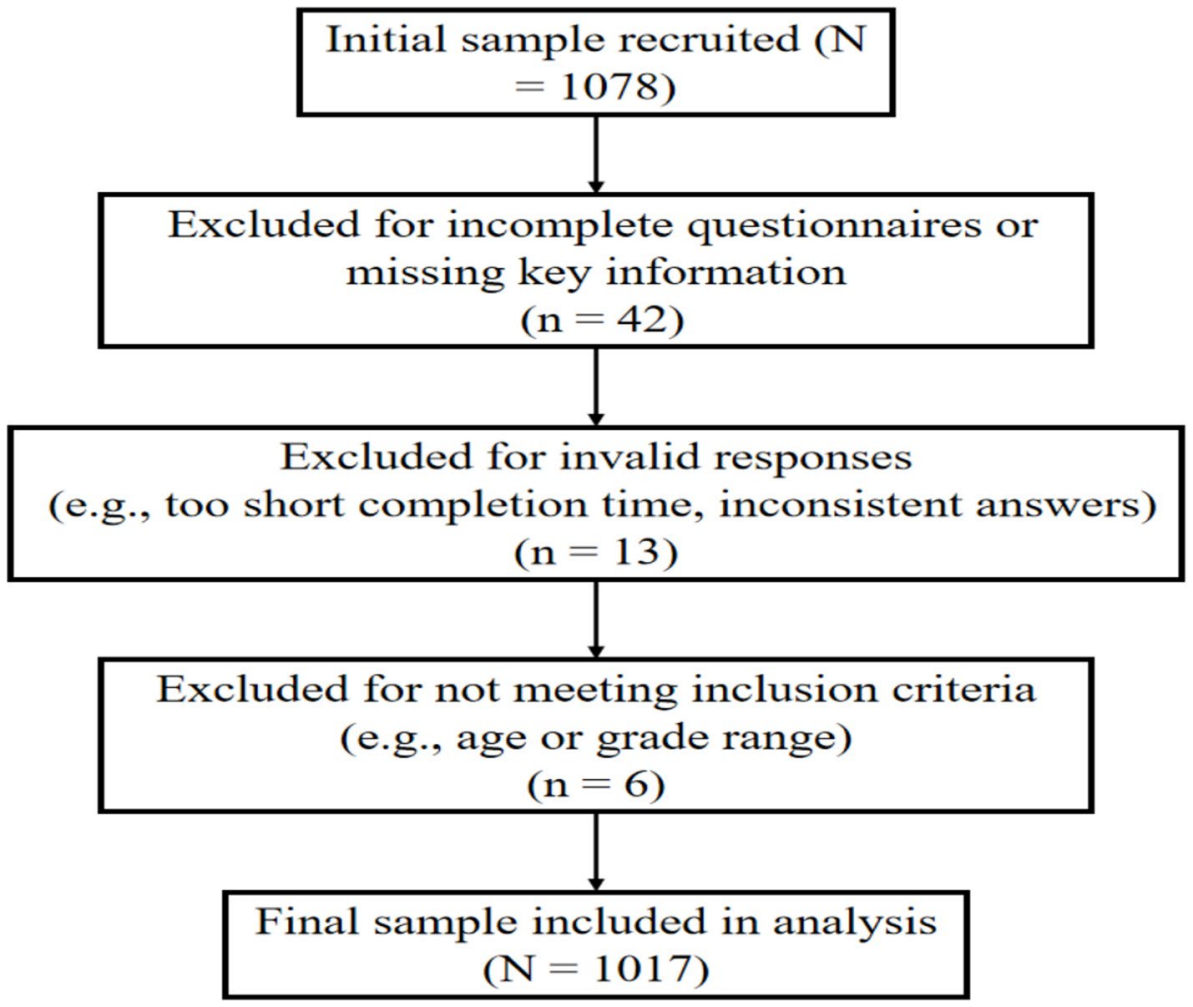
Flowchart of sample inclusion and exclusion.

After excluding invalid responses, 1,017 valid questionnaires were retained, yielding an effective response rate of 94.34%. Participants ranged in age from 12 to 18 years (M = 15.82), with 505 male students (49.66%) and 512 female students (50.34%). The sample included 391 junior high school students (38.45%) and 626 senior high school students (61.55%) (Figure 1).

### 2.2 Procedure

Between March and April 2025, this study employed a stratified random cluster sampling method to collect data from four regions within the Wuling Mountain area: Xiangxi Tujia and Miao Autonomous Prefecture in Hunan Province, Enshi Tujia and Miao Autonomous Prefecture in Hubei Province, Tongren Region in Guizhou Province, and Xiushan Tujia and Miao Autonomous County in Chongqing. From each region, one rural junior high school and one rural senior high school were randomly selected. Within each selected school, one to two classes from grades 7 to 12 were randomly chosen, resulting in a total of 32 classes and 1,078 students who completed the questionnaire (Figure 1).

This study was approved by the Biomedical Ethics Committee of Jishou University (Approval No. JSDX-2023-0034). All data collected were used exclusively for academic research. Prior to data collection, the research team contacted the administrative departments of participating schools to explain the study’s objectives, procedures, and ethical considerations, and obtained written approval from the relevant authorities. In accordance with ethical guidelines for research involving minors, both students and their parents were fully informed about the study’s purpose, procedures, and confidentiality measures. Informed consent was obtained from all student participants and their parents or legal guardians.

### 2.3 Measure

#### 2.3.1 Center for Epidemiologic Studies Depression Scale (CES-D)

The Center for Epidemiologic Studies Depression Scale (CES-D) was used to assess participants’ depressive symptoms over the past week (Wen et al., 2023). The scale consists of 20 items rated on a 4-point Likert scale (1 = “rarely or none of the time,” 4 = “most or all of the time”). Total scores are calculated by summing all item scores, with higher scores indicating more severe depressive symptoms. Following criteria used in previous studies, participants with a total CES-D score < 24 were categorized as No Depression, those with scores between 24 and 28 (inclusive) were classified as having subthreshold depression, and those with scores ≥ 29 were classified as having clinical depression (Su, Zhang, Yu, et al., 2015; Wang and Hanges, 2011). In this study, the CES-D demonstrated good internal consistency, with a Cronbach’s alpha coefficient of 0.88.

#### 2.3.2 Multidimensional scale of perceived social support (MPSSS)

The Multidimensional Scale of Perceived Social Support (MPSSS) (Cosco et al., 2020; Chen et al., 2009), was used to assess adolescents’ perceived social support. The scale consists of 12 items covering three dimensions: family support, peer support, and significant others. Each item is rated on a 7-point Likert scale (1 = “very strongly disagree,” 7 = “very strongly agree”), with higher scores indicating greater perceived social support. In this study, the MPSSS demonstrated good internal consistency, with a Cronbach’s alpha coefficient of 0.86 for the total scale.

### 2.4 Statistical methods

Latent Profile Analysis (LPA), a person-centered statistical approach, was employed to identify unobserved subgroups or ‘profiles’ of individuals based on their patterns of responses across the dimensions of perceived social support (Collins and Lanza, 2010; Muthén and Muthén, 2000). Unlike variable-centered approaches (e.g., linear regression) that examine the average relationship between variables across an entire sample, LPA is uniquely suited to uncover population heterogeneity by identifying distinct clusters of individuals who share similar support characteristics. This approach provides a more nuanced understanding of how social support is experienced differently within the population.

Specifically, LPA was conducted using Mplus 8.3 with the robust maximum likelihood (MLR) estimator, which yields standard errors and chi-square tests (χ^2^ test) robust to the non-normality of observed variables. To determine the optimal number of latent profiles, we estimated a series of models (from one to five profiles) and selected the best-fitting model based on a combination of statistical criteria and theoretical interpretability. These criteria included (Nylund-Gibson and Choi, 2018): (1) lower values for the Akaike Information Criterion (AIC), Bayesian Information Criterion (BIC), and sample-size adjusted BIC (aBIC) indicating better model fit; (2) significant Lo–Mendell–Rubin adjusted Likelihood Ratio Test (LMR-LRT) and Bootstrap Likelihood Ratio Test (BLRT) (*p* < 0.05), suggesting the k-profile model is superior to the k-1 profile model; and (3) a high entropy value (close to 1.0, with > 0.80 being acceptable) indicating high classification accuracy.

Following the identification of the latent profiles, descriptive statistics (frequencies, percentages) were calculated using SPSS 26.0, and group differences were examined using the chi-square test. Subsequently, a multinomial logistic regression analysis was conducted to assess the association between depressive symptom status (the outcome) and the identified latent profiles of perceived social support (the predictor). Demographic variables that were significant in univariate analyses were included as covariates. To ensure the robustness of our model, we performed preliminary checks for multicollinearity and data separation; no issues were detected. A significance level of *α* = 0.05 was adopted for all statistical tests.

To verify the stability and generalizability of the identified latent profiles, a sensitivity analysis was conducted. Given that LPA solutions can sometimes be driven by sampling idiosyncrasies or local maxima rather than true population heterogeneity, it is crucial to assess whether the extracted profiles are robust to sampling variations. We employed a bootstrap LPA approach (with 1,000 resamples) because it allows for a rigorous examination of model stability without reducing the effective sample size, which is superior to split-half validation methods. In each resample, the number of profiles and the patterns of conditional means were re-estimated to determine if the four-profile structure consistently emerged.

## 3 Results

### 3.1 Common method bias test

As this study relied on self-reported data, the possibility of common method bias (CMB) was considered. To assess its impact, Harman’s single-factor test was conducted (Zhou and Long, 2004). The results revealed that five factors had eigenvalues greater than 1, and the first factor accounted for 29.49% of the total variance—below the commonly accepted threshold of 40%. This indicates that no substantial common method bias was detected.

### 3.2 LPA results and labeling of adolescents’ perceived social support

Using the 12 items of the MPSSS as observed variables, LPA was conducted to identify potential subgroups. Six latent profile models were sequentially estimated, and model fit indices were examined to determine the optimal solution. The results showed that AIC, BIC, and aBIC values decreased progressively with the addition of classes. Notably, the four-class model (Model 4) had the highest entropy value, and the LMR and BLRT tests were both statistically significant (*p* < 0.05) across all class comparisons (Table 1). Therefore, Model 4 was selected as the best-fitting model.

**Table 1:** Fit indices for LPA of perceived social support.

Model	AIC	BIC	aBIC	Entropy	LMRT (*p*)	BLMR (*p*)	Class probabilities
1	50647.948	50766.138	50689.912				
2	39240.490	39422.701	39305.186	1.000	<0.001	<0.001	0.56/0.44
3	36952.066	37198.296	37039.492	0.972	<0.001	<0.001	0.29/0.28/0.43
4	34484.633	34794.884	34594.790	0.989	<0.001	<0.001	0.10/0.30/0.16/0.44
5	33910.453	34282.723	34043.341	0.981	0.057	0.061	0.22/0.09/0.16/0.44/0.09
6	33407.566	33845.857	33563.185	0.976	0.041	<0.001	0.08/0.10/0.16/0.15/0.07/0.44

AIC, Akaike information criterion; BIC, Bayesian information criterion; aBIC, sample-size adjusted BIC; LMR, Lo–Mendell–Rubin likelihood ratio test; BLRT, bootstrap likelihood ratio test.

**Figure 2:**
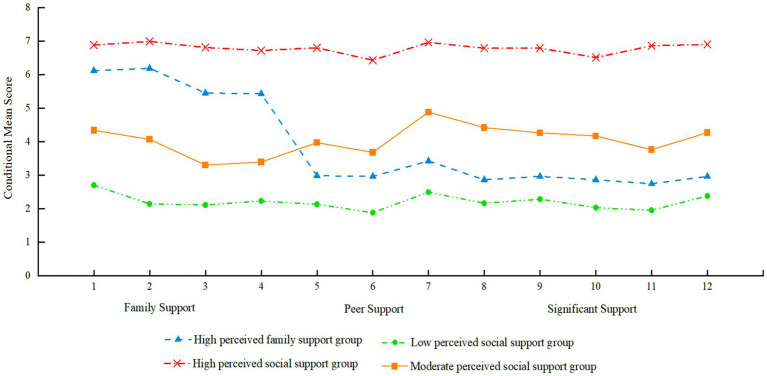
LPA of perceived social support: Four-class solution.

The four latent classes exhibited distinct patterns in the conditional means across the 12 items representing the three dimensions of perceived social support. Class 1 showed the highest conditional means across all three dimensions—family support, peer support, and support from others—and accounted for 43.7% of the total sample; based on its scoring profile, this class was labeled the “High perceived social support” group. Class 2 demonstrated relatively high scores in family support but significantly lower scores in peer support and support from others, comprising 16.2% of the participants, and was thus labeled the “High perceived family support” group. Class 3 displayed moderate scores across all three dimensions and made up 30.4% of the sample, leading to its classification as the “Moderate perceived social support” group. Class 4 had the lowest scores on all dimensions and accounted for 9.7% of the participants; it was designated as the “Low perceived social support” group (Figure 2).

The bootstrap sensitivity analysis confirmed the robustness of the four-profile solution. Across 1,000 resamples, the four-profile model was consistently identified as the optimal solution (reproduction rate > 95%). Furthermore, the conditional mean patterns of the indicator variables in the resampled solutions closely mirrored those of the original full-sample solution. The consistent reproduction of the distinct profiles (high support, high family support, moderate support, and low support) suggests that the identified latent structure is stable and not an artifact of sampling error.

### 3.3 Demographic characteristics of adolescents’ perceived social support patterns

To examine the influence of demographic variables on the four latent classes of perceived social support among adolescents, chi-square tests were conducted to compare the distribution differences across demographic factors. Furthermore, multinomial logistic regression was employed to assess the predictive effects of gender and school stage on class membership. Chi-square test results indicated significant differences in the distribution of perceived social support classes by gender [χ^2^(3) = 386.391, *p* < 0.001] and school stage [χ^2^(3) = 10.296, *p* = 0.016], as shown in Table 2. Using the “Low perceived social support” group (Class 4) as the reference category, multinomial logistic regression analysis revealed that both gender and school stage significantly predicted class membership.

**Table 2:** Demographic characteristics across perceived social support profiles among adolescents.

Characteristic	Low perceived social support group	Moderate perceived social support group	High perceived family support group	High perceived social support group
Overall proportion	98 (9.7%)	310 (30.4%)	165 (16.2%)	444 (43.7%)
Gender				
Male	78 (79.6%)	276 (89.0%)	25 (15.2%)	126 (28.4%)
Female	20 (20.4%)	34 (11.0%)	140 (84.8%)	318 (71.6%)
χ^2^	386.391			
*P*	<0.001			
OR (95%CI)	1 (ref)	0.48 (0.26–0.88)*	9.81 (5.74–16.75)***	21.76 (11.34–41.77)***
School level				
Junior high	23 (23.5%)	125 (40.3%)	66 (40.0%)	177 (39.9%)
Senior high	75 (76.5%)	185 (59.7%)	99 (60.0%)	267 (60.1%)
χ^2^	10.296			
*P*	0.016			
OR(95%CI)	1 (ref)	0.45 (0.27–0.76)**	0.47 (0.26–0.85)*	0.47 (0.28–0.80)**

OR, odds ratio; CI, confidence interval; ref, reference group; χ^2^, Pearson’s Chi-squared test statistic.*p < 0.05; **p < 0.01; ***p < 0.001.

In terms of gender, compared to boys, girls were significantly more likely to be in the “High perceived social support” group [OR = 21.76, 95% CI (11.34–41.77), *p* < 0.001]. To examine robustness of the High vs. Low comparison, we applied Firth’s penalized logistic regression, which yielded an adjusted estimate of OR = 9.87 (95% CI: 3.12–31.25). The effect remained statistically significant, though attenuated compared with the original estimate. Girls were also significantly more likely to be in the “High perceived family support” group [OR = 9.81, 95% CI (5.74–16.75), *p* < 0.001] and significantly less likely to be in the “Moderate perceived social support” group [OR = 0.48, 95% CI (0.26–0.88), *p* < 0.05]. These results suggest that girls generally tended to perceive higher levels of social support.

Regarding school stage, compared to junior high school students, senior high school students were significantly less likely to be in the “Moderate perceived social support” group [OR = 0.45, 95% CI (0.27–0.76), *p* < 0.01], the “High perceived family support” group [OR = 0.47, 95% CI (0.26–0.85), *p* < 0.05], and the “High perceived social support” group [OR = 0.47, 95% CI (0.28–0.80), *p* < 0.01]. These findings suggest an overall decline in perceived social support among senior high school students, likely due to increased academic pressure and developmental demands.

### 3.4 The relationship between different depressive symptoms and perceived social support in adolescence

To explore the relationship between different levels of depressive symptoms and perceived social support during adolescence, demographic variables found to be significant in univariate analyses were included as control variables. The four latent classes of perceived social support served as independent variables (with the “Low perceived social support” group as the reference), and depressive symptom categories were the dependent variables (with “Depression” as the reference). Multinomial logistic regression analysis showed that, compared with the “Low perceived social support–Depression” group, the “Moderate perceived social support” group acted as a protective factor against both no depression and subthreshold depression during adolescence (*B* = 1.759 and 0.975, respectively, *p* < 0.01). Both the “High perceived family support” group and the “High perceived social support” group were protective factors for the no depression condition in adolescence (*B* = 2.133 and 1.581, respectively, *p* < 0.01), with the “High perceived family support” group showing a particularly strong protective effect against no depression (OR = 8.442). Detailed results are presented in Table 3.

**Table 3:** Multinomial logistic regression of depressive symptoms and latent classes of perceived social support during adolescence.

Latent profile group	Depressive symptoms status	*B*	Standard error	OR	OR value 95%CI	*P*
Moderate perceived social support	No depression	1.759	0.302	5.807	3.312–10.499	<0.001
	Subthreshold depression	0.975	0.358	2.650	1.314–5.342	0.006
High perceived family support	No depression	2.133	0.371	8.442	4.082–17.460	<0.001
	Subthreshold depression	0.038	0.514	1.038	0.379–2.842	0.941
High perceived social support	No depression	1.581	0.274	4.860	2.842–8.314	<0.001
	Subthreshold depression	0.288	0.339	1.333	0.686–2.593	0.396

B, unstandardized regression coefficient; SE, standard error; OR, odds ratio; CI, confidence interval; P, *P*-value.

## 4 Discussion

This study employed LPA to identify distinct latent classes of perceived social support among adolescents. Furthermore, it examined the predictive effects of demographic variables (gender and school stage) and depressive symptoms on class membership. The findings revealed heterogeneity in adolescents’ perceived social support, offering new insights into understanding adolescent mental health.

### 4.1 Latent classes of perceived social support during adolescence

The LPA results identified four distinct latent classes of perceived social support among adolescents: the High perceived social support group, the High perceived family support group, the Moderate perceived social support group, and the Low perceived social support group, with significant differences in social support scores across various dimensions among these groups. This finding indicates that adolescents’ perception of social support is not unidimensional but shows marked individual variability (Thoits, 2011).

First, the High perceived social support group, accounting for 43.7% of adolescents, reported high levels of support from family, peers, and other sources, reflecting a comprehensive and balanced social support network. Studies suggest that adolescents in this group typically demonstrate stronger psychological resilience and positive coping strategies, enabling them to effectively utilize multiple channels for emotional comfort and tangible assistance (Yang et al., 2024; Fergus and Zimmerman, 2005). This multi-source support pattern provides a multilayered protective barrier, effectively reducing the risk of mental health problems (Uchino, 2006).

Second, the High perceived family support group, comprising 16.2% of adolescents, mainly experienced strong family support, whereas support from peers and other sources was relatively low (Zhao et al., 2015). This uneven support structure may reflect differences in family cultural backgrounds, particularly in rural China where the family remains the primary source of reliance for adolescents. Although these adolescents may face certain limitations in peer interactions, the family as the core support system still offers stable and reliable psychological security (Uchino, 2006).

Third, the Moderate perceived social support group, accounting for 30.4%, perceived moderate support across all dimensions, indicating a relatively balanced but not outstanding social support network (Uchino, 2006). Adolescents in this group generally receive basic support from family, peers, and other social relationships, though the depth and quality of support may require enhancement. While this moderate level of social support is not ideal, it can still provide some psychological protection, especially when facing mild to moderate stressors (Rueger et al., 2010).

Finally, the Low perceived social support group, making up 9.7% of adolescents, reported low perceived support across all dimensions, suggesting they may concurrently face challenges such as family estrangement, insufficient peer interaction, and lack of social resources (Rose and Rudolph, 2006). This group is considered high-risk for mental health problems; lacking effective buffering support systems, they are more prone to psychological maladjustment when encountering life stress and developmental tasks (Wang et al., 2018). Approximately one-tenth of adolescents in this study belong to this group, highlighting an urgent need for attention and intervention from families, schools, and society. Different types of social support may have varying effects on adolescent mental health, emphasizing the necessity of providing personalized support and interventions tailored to the specific needs of different adolescent groups.

### 4.2 Effects of demographic variables on latent classes of perceived social support during adolescence

Regarding gender differences, the present study revealed a distinct pattern wherein females were significantly more likely than males to belong to the High perceived social support and High perceived family support profiles. These gender differences align with gender socialization theories. Specifically, females are often encouraged to express emotions and maintain close interpersonal relationships (Rose and Rudolph, 2006), which may lead them to be more active in building social networks. Conversely, male adolescents may display lower emotional sensitivity or a reluctance to seek help due to traditional masculinity norms (Gariépy et al., 2016). Therefore, when promoting adolescent social support systems, it is crucial to address the specific needs of male adolescents, encouraging them to challenge traditional gender stereotypes and become more open to seeking support.

Regarding school stage, high school students demonstrated a lower likelihood of belonging to high support groups compared to middle school students, which contrasts with preliminary expectations. This phenomenon may reflect that as academic demands intensify, high school students often experience reduced social interaction time while simultaneously seeking greater autonomy, leading to a perceived distance from external support systems (Wang and Eccles, 2012). Moreover, the heightened pressure of future planning in high school may result in a subjective feeling of insufficient support, particularly when the environment fails to meet their evolving needs (Stice et al., 2004). Thus, educators should recognize that while high schoolers seek independence, they still require robust, albeit different, forms of support.

### 4.3 Relationship between perceived social support and depressive symptoms during adolescence

Our analysis confirmed a robust association between social support profiles and the severity of depressive symptoms. First, adolescents in the Moderate perceived social support group were significantly more likely to be free of depressive symptoms compared to the Low support group. This suggests that even moderate levels of social support serve as a substantial buffer against depression (Gariépy et al., 2016). This finding holds important practical implications: ensuring a basic threshold of social support can effectively reduce the risk of severe mental health issues, even if comprehensive support is unavailable.

Notably, the High perceived family support group demonstrated the strongest protective effect against clinical depression, surpassing even the High (comprehensive) support group. Although this group reported relatively lower peer support, the intense family support appeared sufficient to compensate for these deficits. This result likely reflects the cultural context of the participants; in Chinese culture, the family remains the central source of resilience and emotional security for adolescents (Zhang and Wang, 2022). While peer relationships become important during adolescence, a strong family foundation appears to be the critical “safety net” preventing severe psychopathology (Uchino, 2006).

Furthermore, while the comprehensive High perceived social support group also exhibited a significant protective effect, the magnitude was slightly lower than that of the family-centric group. This suggests that the quality and source of support (specifically from parents) may be more pivotal than the mere quantity or breadth of support sources in preventing clinical depression among this demographic.

### 4.4 Implications

This study expands current understanding of perceived social support in adolescents by identifying four distinct latent profiles, showing that social support varies considerably among individuals rather than being a single uniform construct. The multidimensional perspective, covering family, peers, teachers, and others, provides a clearer framework for future research on how different types of support relate to adolescent mental health.

Practically, recognizing these profiles allows for more targeted interventions. Adolescents with low overall support may need broad-based programs focusing on family and peer support, while those with uneven support (e.g., high family but low peer support) could benefit from efforts to strengthen peer relationships. Schools and mental health professionals can tailor strategies based on these profiles to better address the specific needs of adolescents, improving prevention and intervention outcomes related to depressive symptoms and resilience.

### 4.5 Limitations and future research

This study has several limitations. First, the cross-sectional design prevents establishing causal relationships between perceived social support and depressive symptoms. Future research could adopt longitudinal tracking designs to explore the dynamic interplay between these variables. Second, the sample was limited to rural adolescents in the Wuling Mountain area, whose regional and cultural particularities may restrict the generalizability of the findings. Third, this study relied on self-report questionnaires, which may be subject to social desirability bias and common method variance. Future studies should consider incorporating objective measurement methods and multi-informant reports to enhance the reliability and validity of assessments.

Despite these limitations, this study holds significant theoretical and practical value. It reveals the heterogeneity in adolescents’ perceived social support and offers new perspectives for understanding adolescent mental health. Future research can be further expanded in the following areas: (1) investigating the relationship between different types of perceived social support and the developmental trajectories of depressive symptoms; (2) examining the differential effects of specific social support content (e.g., emotional support, instrumental support) on depressive symptoms; (3) exploring how individual traits (such as attachment styles and personality characteristics) moderate the protective effects of social support on depression; and (4) developing and validating tailored intervention programs based on social support types to provide more targeted practical guidance for promoting mental health among rural adolescents.

## 5 Conclusion

In conclusion, this study moves beyond a monolithic view of social support by revealing its heterogeneous nature among rural adolescents. By identifying four distinct support profiles, our findings underscore that it is not merely the quantity but the specific pattern of support that shapes mental health outcomes. The powerful protective effect of family-centered support, in particular, highlights a critical leverage point for intervention in this specific cultural context. Ultimately, these person-centered insights provide a more precise roadmap for families, schools, and policymakers to develop targeted strategies that effectively bolster the well-being of at-risk youth.

## Data Availability

The raw data supporting the conclusions of this article will be made available by the authors, without undue reservation.
