# Correction: Latent profiles of perceived social support among adolescents and their relationship with depressive symptoms

**DOI:** 10.3389/fpsyg.2026.1787844

**Published:** 2026-02-17

**Authors:** Tanming Liu, Zeng Zhou, Shiyuan Zhang, Fulan Zhang, Chuqi Yan

**Affiliations:** 1 Department of Physical Education, Central South University, Changsha, China; 2 School of Sport Science, Jishou University, Jishou, China; 3 Melbourne Graduate School of Education, University of Melbourne, Melbourne, VIC, Australia

**Keywords:** adolescents, depressive symptoms, latent profile analysis, mental health, perceived social support

Author **Tanming Liu** was erroneously spelled as **Liu Tanming**.

Author **Ziyi Chen** was erroneously included as an author in the published article. The correct author list reads:

Tanming Liu^1^, Zeng Zhou^1, 2*^, Shiyuan Zhang^3^, Fulan Zhang^2^ and Chuqi Yan^2^

^1^Department of Physical Education, Central South University, Changsha, China

^2^School of Sport Science, Jishou University, Jishou, China

^3^Melbourne Graduate School of Education, University of Melbourne, Melbourne, VIC, Australia

The **Author Contributions** Statement has been corrected to read:

TL: Conceptualization, Data curation, Funding acquisition, Investigation, Writing – original draft, Writing – review & editing. ZZ: Data curation, Methodology, Project administration, Writing – original draft, Writing – review & editing. SZ: Conceptualization, Data curation, Investigation, Methodology, Software, Writing – review & editing. FZ: Formal analysis, Investigation, Methodology, Supervision, Validation, Writing – review & editing. CY: Data curation, Investigation, Software, Writing – review & editing.

The copyright for this article was incorrectly written as:

© 2026 Tanming, Zhou, Zhang, Zhang, Yan and Chen. This is an open-access article distributed under the terms of the Creative Commons Attribution License (CC BY). The use, distribution or reproduction in other forums is permitted, provided the original author(s) and the copyright owner(s) are credited and that the original publication in this journal is cited, in accordance with accepted academic practice. No use, distribution or reproduction is permitted which does not comply with these terms.

The correct copyright is

© 2026 Liu, Zhou, Zhang, Zhang and Yan. This is an open-access article distributed under the terms of the Creative Commons Attribution License (CC BY). The use, distribution or reproduction in other forums is permitted, provided the original author(s) and the copyright owner(s) are credited and that the original publication in this journal is cited, in accordance with accepted academic practice. No use, distribution or reproduction is permitted which does not comply with these terms.

The citation for the article was erroneously written as:

Tanming L, Zhou Z, Zhang S, Zhang F, Yan C and Chen Z (2026) Latent profiles of perceived social support among adolescents and their relationship with depressive symptoms. *Front. Psychol*. 16:1647562. doi: 10.3389/fpsyg.2025.1647562.

The correct citation is Liu T, Zhou Z, Zhang S, Zhang F and Yan C (2026) Latent profiles of perceived social support among adolescents and their relationship with depressive symptoms. *Front. Psychol*. 16:1647562. doi: 10.3389/fpsyg.2025.1647562

The original version of this article has been updated.

