# Supplementary material for: Latent profiles of perceived social support among adolescents and their relationship with depressive symptoms
Source: Front Psychol. 2026 Jan 12;16:1647562. doi: 10.3389/fpsyg.2025.1647562 (PMC12832775; doi:10.3389/fpsyg.2025.1647562)
Supplement: Supplementary file 1 [file Data_Sheet_1.PDF]

Attachment 1：

Center for Epidemiologic Studies Depression Scale (CES-D)

Instructions: Below is a list of some of the ways you may have felt or behaved. Please indicate how often you have felt this way during the past week by checking the appropriate space.

Circle the “ 1” if you **Rarely or none of the time (less than 1 day)**
Circle the “2” if you **Some or a little of the time (1–2 days)**
Circle the “3” if you **Occasionally or a moderate amount of the time (3–4 days)**
Circle the “4” if you **Most or all of the time (5–7 days)**

1. I was bothered by things that usually don't bother me.	1	2	3	4
2. I did not feel like eating; my appetite was poor.	1	2	3	4
3. I felt that I could not shake off the blues even with help from my family or friends.	1	2	3	4
4. I felt that I was just as good as other people.	1	2	3	4
5. I had trouble keeping my mind on what I was doing.	1	2	3	4
6. I felt depressed.	1	2	3	4
7. I felt that everything I did was an effort.	1	2	3	4
8. I felt hopeful about the future.	1	2	3	4
9. I thought my life had been a failure.	1	2	3	4
10. I felt fearful.	1	2	3	4
11. My sleep was restless.	1	2	3	4
12. I was happy.	1	2	3	4
13. I talked less than usual.	1	2	3	4
14. I felt lonely.	1	2	3	4
15. People were unfriendly.	1	2	3	4
16. I enjoyed life.	1	2	3	4
17. I had crying spells.	1	2	3	4
18. I felt sad.	1	2	3	4
19. I felt that people disliked me.	1	2	3	4
20. I could not get “going.”	1	2	3	4

Attachment 2:

Multidimensional Scale of Perceived Social Support

Instructions: We are interested in how you feel about the following statements. Read each statement carefully. Indicate how you feel about each statement.

Circle the "1" if you **Very Strongly Disagree**
Circle the "2" if you **Strongly Disagree**
Circle the "3" if you **Mildly Disagree**
Circle the "4" if you are **Neutral**
Circle the "5" if you **Mildly Agree**
Circle the "6" if you **Strongly Agree**
Circle the "7" if you **Very Strongly Agree**

1.	There is a special person who is around when I am in need.	1	2	3	4	5	6	7	SO
2.	There is a special person with whom I can share my joys and sorrows.	1	2	3	4	5	6	7	SO
3.	My family really tries to help me.	1	2	3	4	5	6	7	Fam
4.	I get the emotional help and support I need from my family.	1	2	3	4	5	6	7	Fam
5.	I have a special person who is a real source of comfort to me.	1	2	3	4	5	6	7	SO
6.	My friends really try to help me.	1	2	3	4	5	6	7	Fri
7.	I can count on my friends when things go wrong.	1	2	3	4	5	6	7	Fri
8.	I can talk about my problems with my family.	1	2	3	4	5	6	7	Fam
9.	I have friends with whom I can share my joys and sorrows.	1	2	3	4	5	6	7	Fri
10.	There is a special person in my life who cares about my feelings.	1	2	3	4	5	6	7	SO
11.	My family is willing to help me make decisions.	1	2	3	4	5	6	7	Fam
12.	I can talk about my problems with my friends.	1	2	3	4	5	6	7	Fri

The items tended to divide into factor groups relating to the source of the social support, namely family (Fam), friends (Fri) or significant other (SO).

To make the latent profile analysis plots more intuitive, the 12 items in Figure 2 were reordered after data collection. Items 1 -4 correspond to Family Support (original items 3, 4, 8, 11), items 5 -8 correspond to Peer Support (original items 6, 7, 9, 12), and items 9 -12 correspond to Significant Other Support (original items 1, 2, 5, 10)

References

Canty-Mitchell, J. & Zimet, G.D. (2000). Psychometric properties of the Multidimensional Scale of Perceived Social Support in urban adolescents. *American Journal of Community Psychology*, *28*, 391-400.

Zimet, G.D., Dahlem, N.W., Zimet, S.G. & Farley, G.K. (1988). The Multidimensional Scale of Perceived Social Support. *Journal of Personality Assessment*, *52*, 30-41.

Zimet, G.D., Powell, S.S., Farley, G.K., Werkman, S. & Berkoff, K.A. (1990). Psychometric characteristics of the Multidimensional Scale of Perceived Social Support. *Journal of Personality Assessment*, *55*, 610-17.

Attachment3：

Supplementary Table 1. Mean ± SD of each item across latent classes of perceived social support

Subscales	Items	High perceived family support group	Low perceived social support group	Moderate perceived social support group	High perceived social support group
**family support**	**1**	6.12±0.68	2.7±1.26	4.34±1.22	6.88±0.36
	**2**	6.19±0.81	2.14±1.01	4.07±1.21	6.99±0.14
	**3**	5.45±1.17	2.11±1.06	3.30±1.31	6.81±0.47
	**4**	5.43±1.11	2.23±1.31	3.39±1.38	6.72±0.60
**peer support**	**5**	2.98±0.40	2.13±0.99	3.97±1.09	6.80±0.47
	**6**	2.96±0.54	1.88±1.09	3.68±1.31	6.43±0.70
	**7**	3.42±0.74	2.49±1.26	4.88±1.21	6.96±0.20
	**8**	2.86±0.56	2.16±1.16	4.42±1.41	6.79±0.44
**significa nt others**	**9**	2.96±0.69	2.28±1.19	4.26±1.27	6.79±0.46
	**10**	2.86±0.64	2.03±1.2	4.17±1.35	6.51±0.62
	**11**	2.74±0.73	1.95±0.98	3.76±1.26	6.86±0.42
	**12**	2.96±0.41	2.38±1.25	4.27±1.06	6.90±0.34

Note: Values are presented as mean ± standard deviation (SD) for each item within each latent class.
